# “Soft” Alkali Bromide and Iodide Fluxes for Crystal Growth

**DOI:** 10.3389/fchem.2020.00518

**Published:** 2020-06-26

**Authors:** Vladislav V. Klepov, Christian A. Juillerat, Kristen A. Pace, Gregory Morrison, Hans-Conrad zur Loye

**Affiliations:** Department of Chemistry and Biochemistry, University of South Carolina, Columbia, SC, United States

**Keywords:** flux crystal growth, alkali halide fluxes, chalcogenides, single crystals, inorganic synthesis

## Abstract

In this review we discuss general trends in the use of alkali bromide and iodide (ABI) fluxes for exploratory crystal growth. The ABI fluxes are ionic solution fluxes at moderate to high temperatures, 207 to ~1,300°C, which offer a good degree of flexibility in the selection of the temperature profile and solubility. Although their main use is to dissolve and recrystallize “soft” species such as chalcogenides, many compositions with “hard” anions, including oxides and nitrides, have been obtained from the ABI fluxes, highlighting their unique versatility. ABI fluxes can serve to provide a reaction and crystallization medium for different types of starting materials, mostly the elemental and binary compounds. As the use of alkali halide fluxes creates an excess of the alkali cations, these fluxes are often reactive, incorporating one of its components to the final compositions, although some examples of non-reactive ABI fluxes are known.

## Introduction

Crystal growth from solution rests on the well-known principle of supersaturation that can be controlled by many factors, such as the solvent nature, polarity, temperature, competing compounds present in the solution, etc. All these parameters can be finely optimized to achieve the formation of good quality crystals, which are necessary for the structural characterization via single crystal X-ray diffraction of a new compound (Kanatzidis and Sutorik, 1995). Solution crystal growth in different organic solvents works well for most organic compounds; however, crystallization of non-ionic extended structures is more challenging and a far less understood process. While ionic compounds and complexes can be easily recrystallized from a polar solvent, such as water or acetonitrile, at room or slightly elevated temperature, more covalent extended structures can only be obtained under conditions when the system has enough energy to overcome the energetic barriers related to breaking and recombination of covalent bonds. Although water can still be employed for crystallization at high temperatures (and often is), preventing water evaporation from a reaction vessel becomes increasingly difficult above ~220–230°C, a temperature at which polytetrafluoroethylene (PTFE) softens and standard hydrothermal techniques can no longer be used. Beyond this temperature, a much more sophisticated apparatus has to be used to be able to use water and other common solvents as a reaction media (Pace et al., 2018).

A convenient alternative to conventional solvents is various inorganic compounds that melt at readily achievable temperatures without reaching their boiling points during the reaction, circumventing the necessity of having expensive closed reaction vessels that can withstand high pressures. One well-known example of a high temperature flux is MoO_3_, which has been widely used for crystallizing various phases, for example, uranium oxides (Juillerat et al., 2019). This compound melts at 795°C, creating a reaction and crystallization medium for the starting materials, and can be slowly evaporated to promote the formation of large single crystals by slow oversaturation of the solution. Another major benefit of using fluxes for crystal growth is the ability to assume a kinetic control over a reaction. High temperature solid state reactions are often carried out at temperatures above 600°C to achieve sufficient diffusion rates. A downside of using high temperatures is the inevitable formation of thermodynamic stable products commonly observed with rather simple binary or ternary compositions (Kanatzidis and Sutorik, 1995). The use of a flux allows for circumventing this limitation as a molten, liquid flux component significantly improves diffusion rates of a reaction by solubilizing the starting materials and therefore products can be obtained at lower temperatures and shorter reaction times. This offers an additional degree of control of the reaction conditions, paving a path toward kinetic products.

There are a number of excellent reviews devoted to flux crystal growth in general (Kanatzidis and Sutorik, 1995; Bugaris and zur Loye, 2012; Liu et al., 2013), the use of selected fluxes (Kanatzidis et al., 2005; Kanatzidis, 2017), and materials that can be obtained using fluxes (Bugaris and Ibers, 2010). Chloride fluxes are among the most universal and widely applied fluxes, and have been extensively used for crystallization of oxides, chalcogenides, and chlorides (Bugaris and zur Loye, 2012). Their heavier counterparts, bromide and iodide fluxes, have attracted much less attention, although they offer a wider reaction temperature range. In this review, we focused on alkali bromide and iodide (ABI) fluxes to summarize and rationalize their application in exploratory crystal growth. We show their application to different systems to reveal most often used approaches and highlight possible future directions in the development of the alkali bromide and iodide flux technique.

## General Considerations

The most important factor that governs processes during flux crystal growth is supersaturation. Ideally, once the starting materials have dissolved in a flux at a peak temperature, the supersaturation is reached when the concentration of the products exceeds their solubility upon decreasing temperature. After crossing the saturation limit, the melt becomes oversaturated and the nucleation process, which usually takes place on the walls of the reaction vessel or the surface of the melt, occurs more rapidly than nuclei dissolution, resulting in some of the nuclei reaching their critical size to become seed crystals that initiate the crystal growth process. According to the classical view on crystal growth, the size of the crystals is a function of how fast the oversaturation occurs, the growth of larger crystals is promoted by slower oversaturation, which favors the growth process over nucleation, while the opposite result, a large number of smaller crystals, can be achieved by rapid oversaturation of the melt. Although there are two dominating factors that affect the rate at which the melt is oversaturated, temperature profile, and the nature of the flux, other factors, such as crucible size and material, have been recently realized as having a great impact on the products of a flux reaction and will be discussed in more detail in the following sections.

### Starting Materials

In order to successfully carry out a flux reaction one must carefully select appropriate starting materials, a flux component, a reaction vessel, and a temperature profile. The starting reagents are most commonly considered first since they largely control the composition of the target product, a flux must be then chosen to dissolve the chosen reactants, a reaction vessel must be chosen that will successfully contain the flux, and lastly a temperature profile must be picked that supports all three of the previous choices.

There are several common approaches to choosing starting materials which include recrystallizing a polycrystalline precursor, reaction of the elements, and binary reagents, although starting material selection is oftentimes a matter of reagent availability. Often, a polycrystalline sample of the desired product (precursor) can be obtained easily by other methods, typically by solid state methods, and then recrystallized in a flux to obtain a single crystalline product (Zeng et al., 2008). In this approach, the choice of flux is essential, as the flux should dissolve the starting material in order to aid in the crystallization; however, the flux must not form stable compounds with the components of the precursor, which is often difficult to achieve since the solvent must break the covalent bonds of the precursor. It is hard to predict which flux will be suitable for this role, especially for alkali halide fluxes, as they contain alkali metal cations that readily incorporate into the final products. More often, different fluxes are probed for suitability for a certain system, as it happens with selection of an organic solvent for organic systems.

Other approaches use fluxes both as a reactant and crystallization medium at the same time. One such approach involves the use of elements as starting materials and has proved useful for the precise control over the reaction composition in a closed system. The downside of using some of the elements is their volatility and difficulties with handling hazardous substances. For example, the use of chlorine or bromine is rather limited in closed systems, such as evacuated fused silica tubes, while chloride/bromide fluxes can offer a safe and convenient source of chlorine/bromine (Ruck and Schmidt, 2003; Yahia et al., 2010; Cortese et al., 2015; Read et al., 2015). Sulfur is another example and has been used in the elemental form in many reactions, although it has a boiling point of ~445°C and builds up pressure beyond this temperature, potentially resulting in tube bursting. To prevent this, longer ramping times are required which allow the starting materials to react with each other, forming non-volatile species. This disadvantage can be overcome by using binary compounds, which circumvents the use of elemental sulfur and reduces the reaction time without a significant risk of tube bursting. The use of binaries is especially efficient for exploratory crystal growth as it offers fast screening of phase space and identification of stable compositions. The downside of using the binary compounds is the occasional difficulty of obtaining binary phases that are not commercially available or binary starting reagents that contain unexpected impurities that alter the outcome of the reaction, with product formation depending on the reagent's lot (Wells et al., 2010). For example, rare earth sulfides are not currently commercially available, except for lanthanum sulfide, and require additional experiments to obtain the pure starting materials (Klepov et al., 2019a).

Recrystallization of materials that were obtained via the solid state route and reactions between elements or binaries are by far the most popular reactions employing fluxes. There are some interesting approaches that are not widely employed, but offer an alternative way of synthesis, which sometimes can save a lot of efforts. For example, Guo, Huang, and coworkers use oxides accompanied by elemental sulfur and elemental boron to obtain sulfide phases (Guo et al., 2009). Boron has a high affinity for oxygen, attacking oxides to form B_2_O_3_ that causes the reaction, which is accompanied by the reduction of sulfur to sulfide, to form a sulfide *in situ*. Coupled with a flux that promotes the reaction and then crystal growth, this approach offers a convenient way for exploratory crystal growth as it allows for using oxides, which usually are more readily available than sulfides or the elements. Another approach is oxidative elimination in the presence of NaI/CsI flux that has recently been employed by Woo et al. (2019). In this reaction, CuI was used as an oxidizer to create an oxidized boron phosphide Na_2_BP_2_ according to the reaction Na_3_BP_2_ + CuI → Na_2_BP_2_ + Cu + NaI. This work is a nice illustration of a precise control over the reaction conditions, guided by PXRD, to form a desired species.

Unlike a solid state reaction, which offers the final product in, ideally, pure form at the end of a reaction, flux crystal growth involves one crucial step after the reaction is done—separation of residual flux from the products. For ABI fluxes, most of the common polar solvents serve this purpose well. Although water is the most common choice to dissolve a flux, most of the products obtained via ABI flux crystal growth are air- or moisture-sensitive, which requires the use of anhydrous organic solvents, such as methanol, ethanol, or DMF. These solvents offer a relatively fast, within hours, removal of a residual flux, with little damage to moisture sensitive crystals. Additional precautions should be taken with air sensitive crystals, and properly degassed and dehydrated solvents should be used.

### Reaction Vessels

Compatibility between the reaction vessel and the flux is among the most important criteria for choosing a reaction vessel. While ABI fluxes are largely compatible with almost any reaction vessel of choice, using a mixed flux that includes a fluoride component will limit the available choices, as fluorides tend to extensively attack fused silica and alumina reaction vessels. Although fluoride fluxes are incompatible with alumina, small amounts of metal fluorides can be tolerated without debilitating damage to the vessel. Some of the syntheses reported for ABI fluxes use metal fluoride starting materials, specifically Schleid's work, and carbon coating the tube, or containing the reaction in an alumina crucible or gold ampule and using the fused silica as secondary containment proved successful in protecting the fused silica tube from the small amount of metal fluoride. Although ABI fluxes are generally considered compatible with fused silica tubes, there are reports on using a bromide flux in a fused silica tube that resulted in Si incorporation from the tube (Lipp et al., 2012). This can be limited by the use of carbon coating, or a secondary container.

Other important factors for choosing reaction vessels are the desired atmosphere, the melting point, and in some cases the surface to volume ratios. Reaction vessels open to atmosphere, for example alumina and platinum crucibles, are primarily used for oxide synthesis, as targeted synthesis of halide, chalcogenide, and pnictide products often limit the amount of oxygen available to the reaction. Evacuated and sealed fused silica and Pyrex tubes can be used for closed systems, where fused silica has a much higher melting point and is more frequently used than Pyrex. The synthesis of the reported chalcogenides, oxychalcogenides, and pnictides, are almost exclusively carried out in evacuated fused silica tubes to exercise control over the incorporation of oxygen. In contrast, many different reaction vessels were used for halide containing products, including open alumina and platinum reaction vessels and sealed fused silica tubes. Additionally, several reported syntheses used He arc-sealed tantalum or niobium ampules. Although not discussed in any ABI flux papers, the surface area to volume ratio of the reaction vessels has been found to play a significant role in alkali chloride/fluoride flux reactions and could be important to consider in further studies using ABI fluxes (Morrison et al., 2016a).

### Temperature Profiles

**Figure 1:**
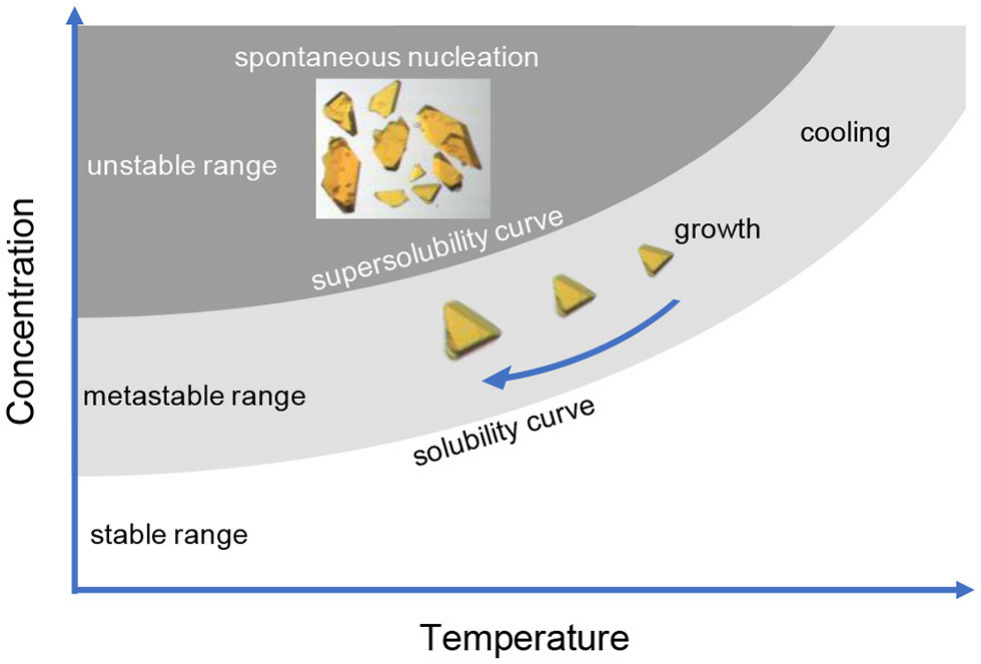
A simplistic representation of crystallization process using solutions. After melting a sufficient amount of starting materials at high temperature (top right corner), the reaction is slowly cooled to achieve a supersaturated solution and promote crystal growth.

For crystal growth to occur, a solution must be sufficiently supersaturated to facilitate nucleation. In molten solutions, it is important to select a flux that is capable of dissolving the reactants and has a substantial change in solubility over the temperature range of interest, otherwise, the nucleated crystals will be re-dissolved and no single crystals will form. The optimal rate of nucleation occurs over a given temperature range which is specific to each system, thus exploratory crystal growth largely focuses on this determination (Figure 1; Bugaris and zur Loye, 2012; Juillerat et al., 2019).

**Figure 2:**
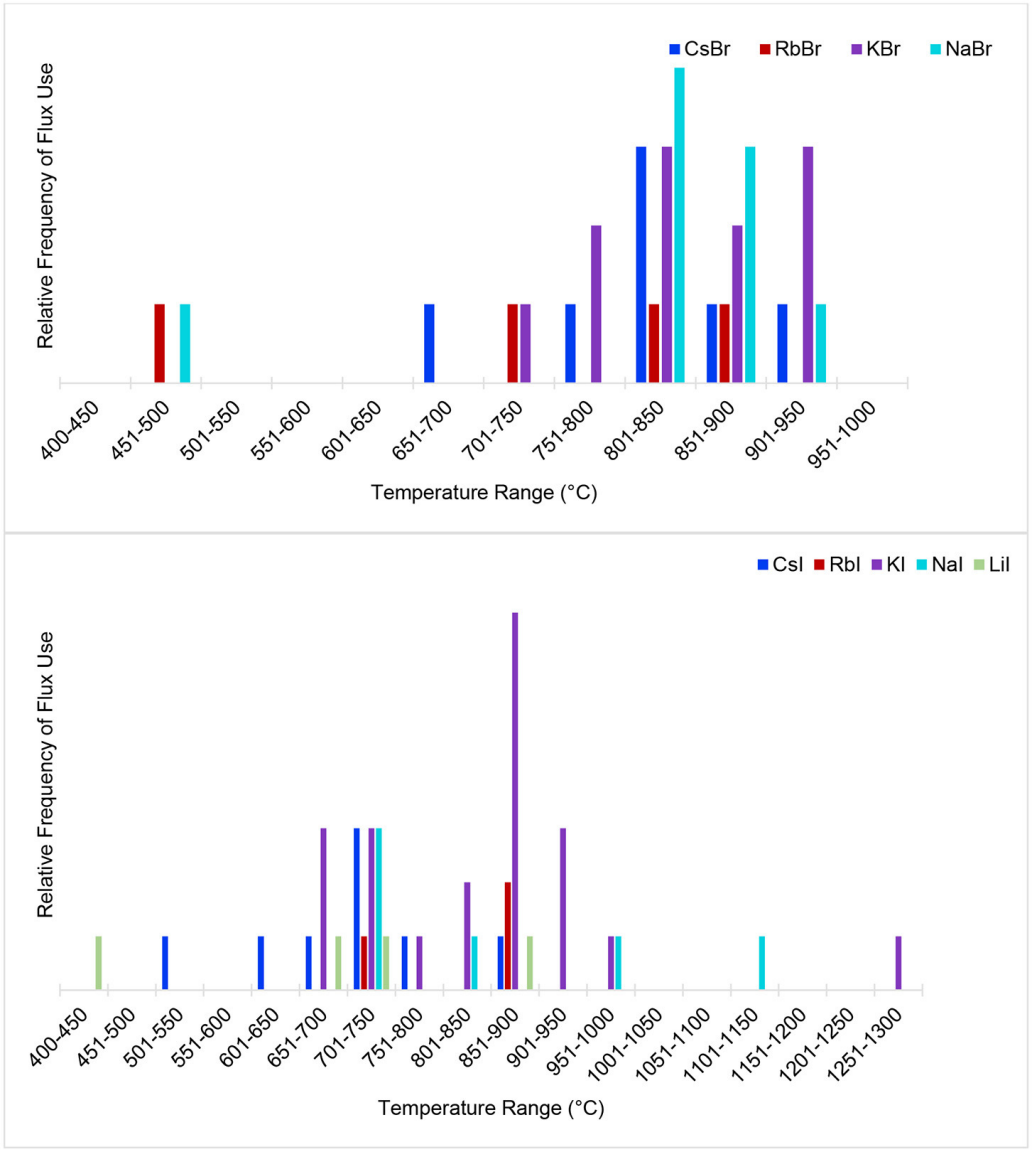
Flux temperature ranges that have been used to obtain the compounds discussed in this review.

Indeed, selecting an appropriate reaction temperature is among the most critical considerations to make when conducting crystal growth experiments. ABI fluxes offer a great deal of versatility when it comes to accessible temperature ranges, and have generally been used over a wide temperature range from ~400 to 1,000°C. The melting points of the ABI fluxes and some selected eutectics are listed in Table 1. An extremely helpful tool in flux selection is the FactSage thermochemical database, which offers a large number of binary salt phase diagrams with the compositions and melting points of eutectic mixtures (Bale et al., 2002, 2009, 2016). Figure 2 shows the temperature ranges used most frequently for the compounds discussed in this review, and demonstrates that reactions involving ABI fluxes have typically been carried out at temperatures 50–200°C higher than the melting point of the flux. There are many examples that indicate a significant influence of the reaction temperature on the resulting crystal morphology; for example, lower temperatures may produce irregularly-shaped crystals while increasing temperature may favor more defined morphologies.

**Table 1:** Melting point of alkali bromide and iodide fluxes and some eutectics with low melting points.

**Salt**	**m.p., **°**C**	**Salt**	**m.p., **°**C**	**Mixtures, molar ratio**	**m.p., **°**C**
LiBr	552	LiI	469	CsI/LiI (34:66)	~207
NaBr	747	NaI	661	KI/LiI (63:37)	~286
KBr	734	KI	681	CsBr/LiBr(37:63)	~281
RbBr	693	RbI	642	NaI/CsI (49:51)	~420
CsBr	636	CsI	621	CsBr/NaBr (58:42)	~458

One important feature of alkali iodide fluxes is their ability to build up pressure of iodine upon heating to high temperatures. This feature can be illustrated by a recent report by Winiarski et al., in which solid state incorporation of CsCl, CsBr, and CsI into Cu_5_O_2_(PO_4_)_2_ was carried out to achieve the formation of the (Cs*X*)Cu_5_O_2_(PO_4_)_2_ (*X* = Cl, Br, and I) salt inclusion phases (Winiarski et al., 2019). While CsCl and CsBr can be obtained by quenching a solid state reaction between the respective alkali halide salt and Cu_5_O_2_(PO_4_)_2_, the iodide analog was obtained by slow cooling to equilibrate the iodine vapors with the sample. The presence of water vapor can increase the amount of iodine formed via the reaction: CsI + H_2_O → CsOH + HI, with subsequent decomposition of HI to the elements (Gouëllo et al., 2018). Considerable work has been carried out to study the thermal behavior of CsI due to the presence of radioactive iodine and cesium, present as CsI in nuclear reactors (Gouëllo et al., 2018).

There has also been a wide range of reaction dwelling times reported for ABI fluxes, ranging from no dwell to dwell periods of up to 5 weeks. In general, there does not seem to be much correlation between the flux used and the selected dwell times, though in many cases, increasing dwell times results in larger crystals. Similarly, cooling rates of ~1–20°C per hour over a range of about 200–500°C below the dwell temperature have been identified as suitable slow-cooling periods, although many of the discussed compounds were prepared with no slow-cooling, thus it is not clear whether or not this is a necessary step in every synthesis. It is also worth noting that several of the synthetic procedures used to prepare the compounds discussed in this review include a multi-step temperature profile, in which the initial synthesis step involves slowly ramping to a given temperature and possibly dwelling at that temperature for some time, followed by quickly heating to the reaction temperature. This is a useful technique for reactions which contain reagents prone to decomposition or those which require a reaction of the starting materials prior to dissolving the reagents in the flux; for example, the decomposition of NH_4_H_2_PO_4_ into a P_2_O_5_ flux or the pre-reaction of elemental sulfur or selenium with other reagents present in the reaction.

## Materials Obtained Using ABI Fluxes

**Figure 3:**
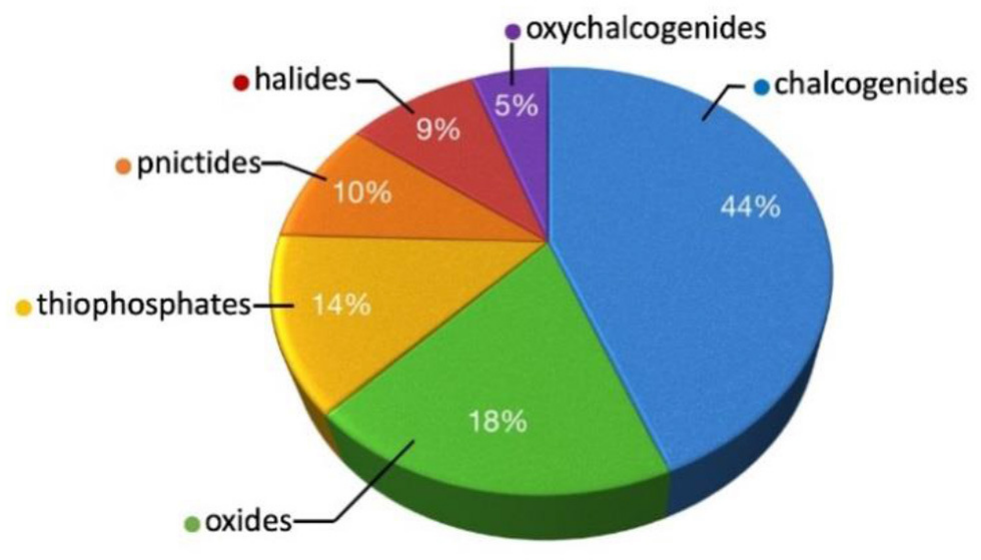
An approximate distribution of the phases obtained using ABI fluxes.

By far the most numerous materials obtained from ABI fluxes are chalcogenides and oxychalcogenides, which comprise almost 50% of all phases (Figure 3). One of the main reasons for that is the suitability of the soft Pearson bases, bromide and iodide, to dissolve and provide a reaction medium for “soft” chalcogenides, whereas “harder” fluoride and chloride fluxes are more suitable for the oxide systems. There is however no strict boundary between the application of both types of fluxes and sometimes they can be used interchangeably. Another reason is that many research groups, once they found suitability of a certain flux for a system, tend to apply the same flux to all similar systems, which can also result in preference of some fluxes over other, although it does not necessarily mean that these phases could not be obtained by other fluxes.

## Oxides

The ABI fluxes have proved successful for the exploratory crystal growth of new complex oxides, where the bromides have received more attention compared to the iodides. In addition to exploratory crystal growth, the ABI fluxes have been used to recrystallize polycrystalline powders, control crystal morphology, and control structure compositions. The reactions generally consist of reacting the appropriate oxides, and in some cases the metal salts, with the ABI containing flux and heating the mixture to between 600 and 900°C for times ranging from a few hours to several days. CsBr was the most frequent flux used in oxide synthesis; in addition, several mixed fluxes were used including KCl/KI, NaBr/KBr, NaBr/CsBr, KI/KNO_3_, and the very complex NH_4_Cl/AlF_3_/NaCl/KBr/H_3_BO_3_ mixture.

CsBr has been extensively used as a flux by the Schleid group for the single crystal growth and structure determination of several families of rare earth selenates and silicates, namely MF(SeO_3_) (M = Sc, Y, Ho-Lu), Ho_3_(SeO_3_)_4_, Sc_2_O_2_(SeO_3_), Er_3_F(SiO)_4_(SeO_3_)_2_, and Pr_5_(SiO_4_)_2_(SeO_3_)_3_ (Wickleder et al., 2002; Lipp and Schleid, 2007, 2008a,b,c, 2009; Lipp et al., 2012, 2013; Chou et al., 2014; Greiner et al., 2017). The MF(SeO_3_) family features an unusual pentagonal bipyramidal coordination environment for the rare earth cation. Generally, the respective rare earth sesquioxide, rare earth fluoride, and SeO_2_ were mixed in a 1:1:3 ratio with an excess amount of CsBr and heated in a carbon coated silica ampule at 700–850°C for 5–7 days. In the case of the silicates, the silica tube reaction vessel acts as the SiO_2_ source due to the reactive nature of fluorides toward the silica. Schleid used alumina crucibles or gold ampules contained within the silica tubes to protect the tube from the reactive fluoride.

The ABI fluxes have also been used to control the crystal morphology of several oxides such as NaNd(MoO_4_)_2_ (Liu et al., 2016), KEu(MoO_4_)_2_ (Wu et al., 2013), Li_2_NiPO_4_F (Yamada et al., 2018), and NaNbO_3_ (Hamilton et al., 2020). In each case the identity of the flux or the amount of flux played a significant role in the crystal morphology. For KEu(MoO_4_)_2_, stoichiometric amounts of K_2_CO_3_, Eu_2_O_3_, and MoO_3_ were mixed with excess amounts of KCl or KBr flux and heated at 700–850°C for 2–6 h in an alumina crucible. As the temperature increases, the morphology of the isolated crystals changes from undefined to rod-like and ultimately to octahedral. Reactions at 750°C were determined to be best for rod shaped crystals and longer dwell times produced larger crystal sizes, while KBr was preferred over KCl, as it produced crystals more uniform in size (Wu et al., 2013).

Similarly, in the synthesis of NaNd(MoO_4_)_2_, the bromide flux resulted in a smaller distribution of crystal sizes. Crystals of NaNd(MoO_4_)_2_ were obtained with mixing Na_2_CO_3_, Nd_2_O_3_, and MoO_3_ in stoichiometric amounts and adding excess amounts of NaCl or NaBr flux and heating the mixture in an alumina crucible at 750–900°C for 10 min to 6 h. In addition to the flux identity, the temperature and dwell time were important variables for control over the morphology of the crystals. Using a lower dwell temperature of 750°C produced crystals with no defined morphology, and as the temperature was increased more defined octahedral crystals were obtained. Larger crystals can be obtained with longer dwell times, and the enhanced size of the crystals resulted in an increase of the emission peaks of the luminescent compound (Liu et al., 2016).

**Figure 4:**
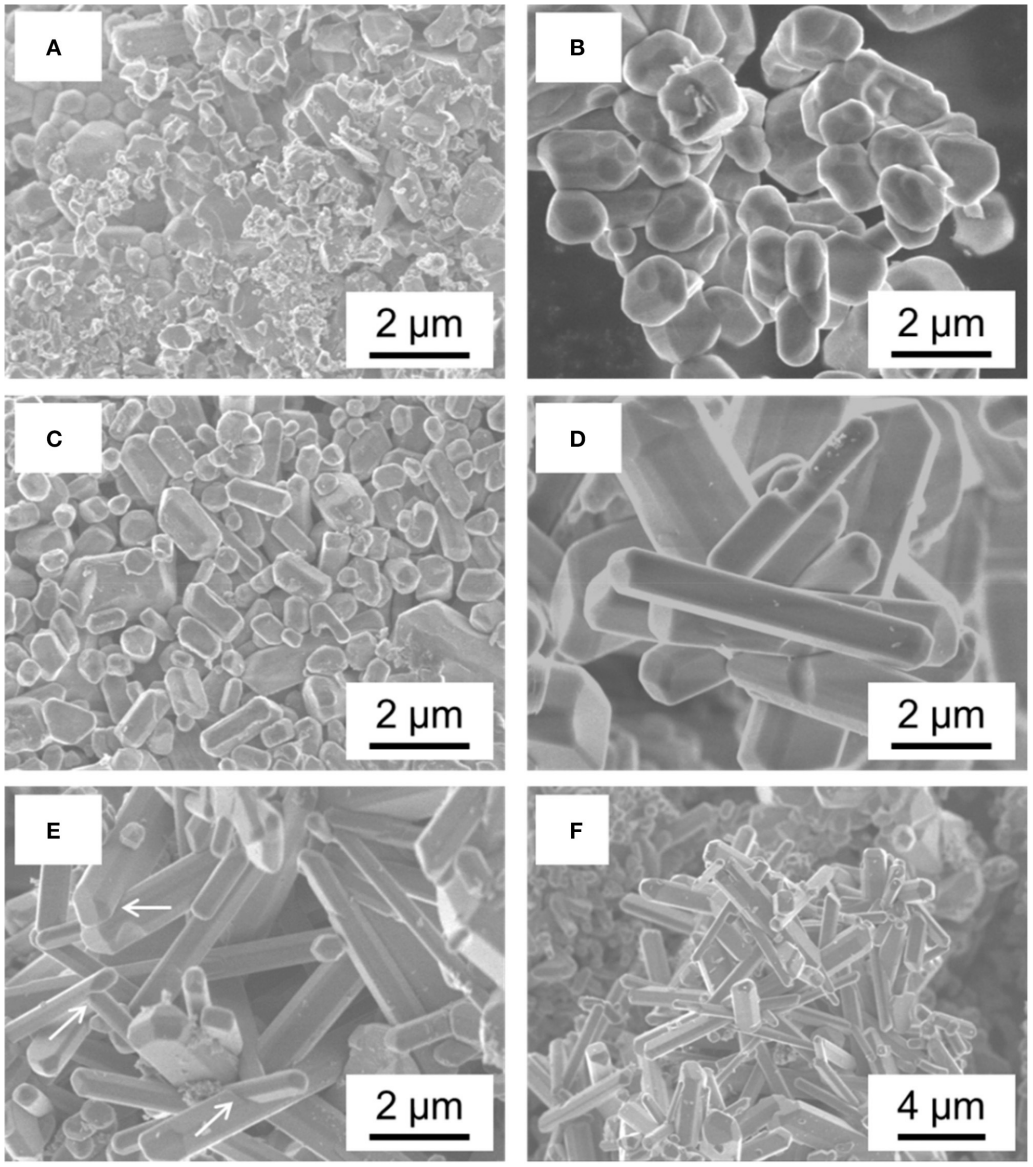
SEM images of Li_2_NiPO_4_F grown from KCl-KI fluxes showing the progression of crystal morphology as the mol % of reactants to flux decreases **(A)** 100, **(B)** 80, **(C)** 50, **(D)** 20, and **(E)** 10 mol %. **(F)** Lower magnification image of **(E)**. Reproduced from Yamada et al. (2018) with permission of American Chemical Society.

Crystals of Li_2_NiPO_4_F were grown from mixtures of LiCO_3_, NiO, NH_4_H_2_PO_4_ that were preheated at 400°C for 3 h before adding LiF and excess KCl/KI flux and heating the mixture to 600°C for 1 h. The morphology of the Li_2_NiPO_4_F crystals was sensitive to the ratio of the preheated reactants and to the amount of KCl/KI flux (Figure 4). By increasing the amount of the flux, the morphology of the crystals changed from undefined to faceted, to rectangularly shaped, to one dimensional rods (Yamada et al., 2018).

A variety of fluxes were used for the synthesis of NaNbO_3_ in order to determine the influence on particle size and morphology as well as photocatalytic activity. Out of the fluxes used (Na_2_SO_4_, NaF, NaCl, and NaBr), NaBr produced the largest particles with the surface area of the particles was nearly twice as large as the next leading flux; however, photocatalytic activity was much higher in particles grown using Na_2_SO_4_ (Hamilton et al., 2020).

The successful synthesis of Sr_3_MgSi_2_O_8_:Eu^2+^ was also dependent on the ratio of the flux components. Sr_3_MgSi_2_O_8_:Eu^2+^ made by mixing SrCO_3_, MgO, SiO_2_, and Eu_2_O_3_ with varying ratios of a four or five component flux including, NH_4_Cl, AlF_3_, NaCl, KBr, and H_3_BO_3_. Multiple ratios were attempted to find favorable conditions for the doping of Eu^2+^, and also the best conditions for maximized luminescence. The authors concluded that changing the concentrations of NH_4_Cl and H_3_BO_3_ greatly influenced the luminescence of the products, where increasing NH_4_Cl seemed to improve luminescence efficiency, while increasing the amount of H_3_BO_3_ had a negative impact on the efficiency; the relative amounts of NaCl and KBr did not play a significant role (Zhang et al., 2016).

The identity of the alkali halide flux was also used to control the doping of the alkali cation, and thus the oxidation of Mn, in Na and K doped LaMnO_3_. For both Na and K doped LaMnO_3_ systems, La_2_O_3_ and MnCO_3_ were added to the desired flux (ACl, ABr, or AI) and heated at 900, 850, or 750°C, respectively (Shivakumara et al., 2001, 2004). In both systems, the doping of the alkali cation was most extensive in the AI flux, i.e., La_0.85_Na_0.14_MnO_2.97_ (NaCl, 900°C), La_0.88_Na_0.12_Mn_0.96_Na_0.04_O_3_ (NaBr, 850°C), and La_0.84_Na_0.16_Mn_0.93_Na_0.07_O_3_ (NaI, 750°C) and La_0.85_K_0.08_MnO_2.93_ (KCl, 900°C), La_0.89_K_0.07_MnO_2.99_ (KBr, 800°C), and La_0.88_K_0.10_Mn_0.98_O_3_ (KI, 750°C). In the Na system, a small amount of Na was also doped on the Mn site if NaBr or NaI were used. In the K system, the more extensive doping of the K in the case of using a KI flux leads to a higher symmetry structure type. No discussion was given on how temperature influences the incorporation of Na or K, although different temperatures were chosen for the different fluxes.

In the study of the crystal growth of MgFe_2_O_4_, MgAl_2_O_4_, and MgCr_2_O_4_ the identity of the chosen flux was determined to have significant effects on the yield of the reaction. MgFe_2_O_4_, MgAl_2_O_4_, and MgCr_2_O_4_ can be synthesized by mixing MgO and the corresponding M_2_O_3_ with excess NaBr or KBr and heating in a platinum crucible. While both fluxes were effective at producing the desired product, using LiF or LiCl resulted in a higher yield as compared to NaBr and KBr (Yanagida and Atumi, 1967). ABI fluxes are also useful for recrystallizing powder products obtained from traditional solid state routes in order to obtain single crystals large enough for single crystal structure determination. For example, both Cs_2_V_4_O_9_ and Rb_2_V_3_O_8_ crystals were obtained in this manner using a CsBr or RbBr flux. Crystals of Cs_2_V_4_O_9_ were grown by carrying out the solid state reaction with CsVO_3_ and V_2_O_3_ in a sealed Pyrex tube at 550°C for 10 h, followed by mixing the resulting powder with a large excess of CsBr in a fused silica tube and heating at 700°C before slow cooling at 6°C/h to 470°C (Liu and Greedan, 1995). The crystal growth of Rb_2_V_3_O_8_ was carried out in a similar manner, where stoichiometric ratios of RbVO_3_, V_2_O_3_, and V_2_O_5_ are mixed and heated at 550°C overnight in a sealed tube, then the resulting powder is added to excess RbBr flux and heated in a sealed fused silica tube to 750°C and slowly cooled to 500°C at 6°C/h (Liu and Greedan, 1995).

A number of complex metal oxides have been obtained from ABI fluxes, where the ABI flux plays an important role of providing alkali cations that incorporate into the final structure. Crystals of Cs_2_Cu_3_P_4_O_10_ were grown from CuO, P_2_O_5_, and CsI flux in an evacuated fused silica tube heated at 700°C for 2 days followed by slow cooling to 300°C at 6°C/h (Sanjaya Ranmohotti et al., 2006). Similarly, NaCuAsO_4_ was grown from the combination of As_2_O_5_, CuO, CuBr_2_, and Cs_2_O in an excess of eutectic NaBr/CsBr flux. Carbon coated silica ampules were used as the reaction vessel and heated to 300°C for 1 day, then 650°C for 4 days followed by slow cooling to 450°C at 2.5°C/h (Ulutagay-Kartin et al., 2003). To obtain crystals of Ba_2_K_2_Te_2_O_9_, Ba(H_4_TeO_6_) was added in a 1:5 weight ratio with KNO_3_/KI flux and heated in a platinum crucible to 500°C, held for 4 days, and slow cooled to room temperature in 12 h (Weil, 2018). The layered oxide, Na_0.27_CoO_2_, can be prepared by mixing CaC_2_O_4_·2H_2_O with 10 times excess NaI and heating in an alumina crucible at 750°C for 24 h (Shivakumara and Hegde, 2003). Lastly, Cs_3_(UO_2_)_2_(PO_4_)O_2_ can be obtained by loading a platinum crucible with (UO_2_)_3_(PO_4_)_2_(H_2_O)_4_ and CsI flux and heating to 750°C for 10 h followed by slow cooling at 7°C/h to room temperature. The unique 3D framework contains larger pores filled with Cs cations (Yagoubi et al., 2013). On the contrary, the synthesis of Bi_2_(Sr_1−x_Ca_x_)_3_Cu_2_Oy gives an example of an unreactive flux. This composition was obtained from dissolving stoichiometric ratios of Bi, Sr, Ca, and Cu nitrates in water and heating up to 500°C and dwelling for 20 h before adding 75% by weight KBr flux and heating in a platinum crucible at 3 h followed by heating to 850 or 880°C and slow cooling to 700°C at 1°C/h (Shishido, 1990).

## Halides/Oxyhalides

There are about half as many halide containing phases grown from ABI fluxes as compared to oxide phases and the structures of the halide containing phases are highly varied and include simple binary phases, perovskite related, high order extended structures, and organic containing extended structures. As compared to the oxides, whose syntheses had a wide variety of mixed fluxes reported, almost all the oxyhalides are grown from single component melts.

Among the reported binary phases are Hf_0.86_I_3_ and TaBr_2.94_ (Habermehl et al., 2010; Beekhuizen et al., 2011). Black rod-shaped crystals of Hf_0.86_I_3_ were produced by reducing HfI_4_ with aluminum at 850°C in the presence of NaI flux contained in a sealed tantalum vessel and held for 16 days before slow cooling to room temperature at 5°C/h. The structure is composed of hexagonal close packed layers of iodide ions where 5.16 out of the 6 octahedral holes are filled by Hf ions (Beekhuizen et al., 2011). TaBr_2.94_ completes the five-member family of binary tantalum bromides TaBr_5_, TaBr_4_, Ta_6_Br_15_, and Ta_6_B_14_; the TaBr_2.94_ structure is distantly related to the perovskite structure type. TaBr_2.94_ was obtained by the reduction of TaBr_5_ with the wall of the tantalum reaction vessel at 500°C with NaBr or RbBr flux (Habermehl et al., 2010).

The ternary phases include Cs_2_AuBr_6_, Cs_2_AuI_6_, Y_16_Br_24_Ir_4_, and the derivatives of the Ba_7_F_12_Cl_2_ structure. Single crystals of the distorted perovskites, Cs_2_AuBr_6_ and Cs_2_AuI_6_, were obtained from slow heating mixtures of Au, Br_2_ or I_2_, and CsI in alumina crucibles sealed inside fused silica tubes at 630 and 550°C, respectively over 42 h and dwelling for 10 h. After heating, the reactions were cooled at 5°C/h (Riggs et al., 2012). Y_16_Br_24_Ir_4_ was obtained in a good yield using Ir metal, YBr_3_, and a RbI or KI flux and heating in a sealed Nb tube at 900°C for 5 weeks (Steinwand and Corbett, 1996). Ba_6.668_Ca_0.332_F_12_Br_2_ and the Ba_7−x_Ca_x_F_12_(Cl_y_Br_1−y_)_2_ family both adopt the Ba_7_F_12_Cl_2_ structure and were obtained from the appropriate alkaline earth halides and a NaBr or NaCl/NBr flux, respectively. The reactions were contained in covered platinum reaction vessels and heated to 900°C and cooled to 600°C in 3 h (Frühmann et al., 2004).

Further examples demonstrating the versatility of ABI fluxes include the novel lithium europium and strontium carbodiimides, LiSr_2_(NCN)I_3_, LiEu_2_(NCN)I_3_, and LiEu_4_(NCN)_3_I_3_, which resulted from a reaction of EuI_2_, NaCN, NaN_3_, and LiI (Liao et al., 2004a). The reactions were contained in sealed tantalum ampoules within sealed fused silica tubes and heated to 880°C [or 700°C for LiEu_4_(NCN)_3_I_3_] for 3 days and cooled at 6°C/min to room temperature. Both Eu containing compositions were obtained using the same ratio of reactants and the product identity was controlled by adjusting the temperature (Liao et al., 2004a). The presence of divalent europium in the structure was confirmed by magnetic measurements, with effective magnetic moments of 8.00(7) and 7.65(5) μ_B_ per Eu, respectively, within the expected range of magnetic moments for Eu^2+^. LiSr_2_(NCN)I_3_ features a unique extended structure with empty tetrahedral Sr_4_ entities (Liao et al., 2004b). Crystals of the M_6_N_3_S_4_Br (M = La – Nd) compounds were grown from oxidizing the rare earth metal with sulfur and NaN_3_ with the corresponding MBr_3_ in the presence of a NaBr flux. The reactions were heated at 850°C for 7 days in evacuated silica tubes (Lissner and Schleid, 2002).

The reported synthetic conditions for obtaining halides, so far, have not used any metal oxide starting materials, but rather used metals in their elemental forms, likely to prevent the inclusion of O in the resulting structures. Two of the three oxyhalides use oxide precursors, while the oxygen source for ((C_2_)_2_O_2_Dy_14_)I_24_ is not discussed. La_3_OBr(AsO_3_)_2_ was synthesized from heating stoichiometric mixtures of NH_4_H_2_AsO_4_, NH_4_Br, and La_2_O_3_ in platinum crucibles at 500°C for 6 h and 850°C for 60 h before adding 20 equivalents of NaBr/KBr flux and heating at 900°C for 72 h and slow cooling at 10°C/h to room temperature (Yahia et al., 2010). In the synthesis of the oxyhalide, CsSm_21_(SeO_3_)_24_Br_16_, SmOBr and SeO_2_, were reacted in a CsBr flux and heated at 797°C and slow cooled to 570 K at 5°C/h (Ruck and Schmidt, 2003). Crystals of the oxyhalide ((C_2_)_2_O_2_Dy_14_)I_24_, were synthesized from DyI_3_, Dy powder, graphite powder, and NaI flux using a He-arc sealed tantalum reaction vessel heated at 1,000°C for 10 days; however, the source of O in the structure was not discussed (Daub and Meyer, 2010).

## Chalcogenides

### Binaries

ABI fluxes were employed to synthesize or recrystallize several binary chalcogenide compounds. In order to obtain new, non-linear optical materials, Zhang et al. performed a reaction between Ga_2_O_3_, S, and B at 950°C (Zhang et al., 2013). In the absence of a flux, the reaction resulted in monoclinic Ga_2_S_3_, whereas the addition of KI flux favored the formation of a cubic polymorph. Both compounds show a good Second Harmonic Generation (SHG) response and an exceptional laser induced damage threshold (LIDT). These two reactions are good examples of how a flux can divert a reaction pathway and lead to a different product, emphasizing the importance of new flux applications to known systems to discover new phases.

**Figure 5:**
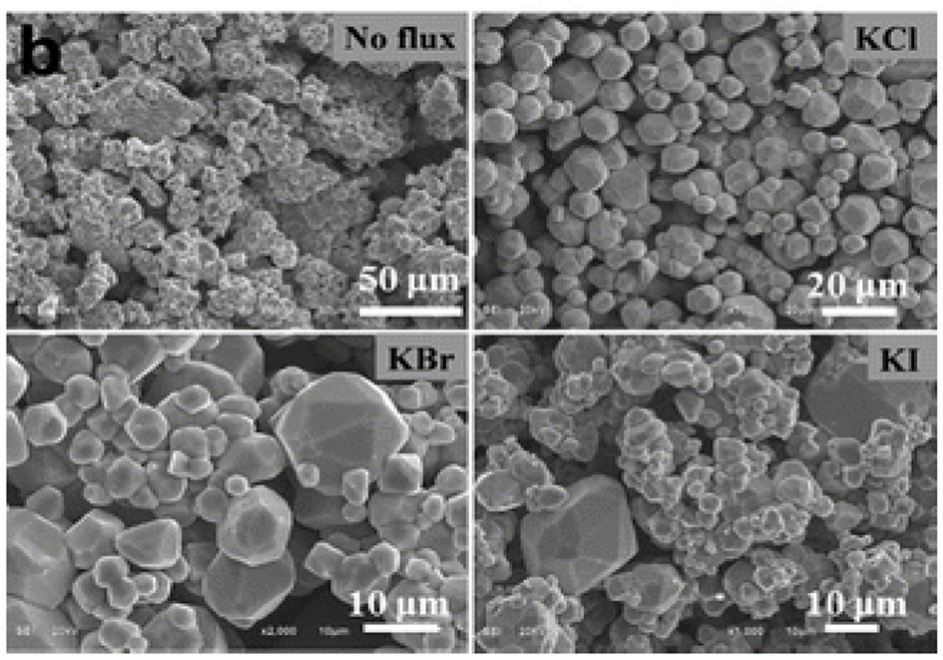
The influence of different fluxes on the size and shape of CoS_2_ particles. Reproduced from Wang et al. (2012) with permission of American Chemical Society.

The application of different fluxes and the use of different flux to reagent ratios can also help fine tune the particle size of binary chalcogenides. For example, ZnS:Cu, Al phosphor particle sizes can be optimized by controlling the identity and the amount of the flux (Kawai et al., 1981). Interestingly, fluxes like NaCl and CaCl_2_ facilitate a fast initial particle growth followed by virtually no further size increase with time, whereas CsCl, KCl, KI, and NaI fluxes behaved in a similar way except they promoted a secondary particle growth step after the plateau time. Another example of a flux controlled particle size synthesis is growth of CoS_2_ particles in KCl, KBr, and KI fluxes (Figure 5; Wang et al., 2012). In the absence of a flux, the reaction between stoichiometric amounts of Co and S resulted in particles with an irregular morphology, while the flux reactions resulted in faceted, polyhedral particles. Among the three fluxes, the KCl flux was found to provide the most uniform sized polyhedra.

Large single crystals of PdS for electrical transport property measurements were grown from a KI flux (Cao et al., 2018). A powder sample of PdS was initially obtained by reacting stoichiometric quantities of the elements at 900°C and then recrystallized from a 20-fold excess of KI at the same temperature, demonstrating the ability of the KI flux to dissolve sulfide materials. The same flux has been used to obtain single crystals of PrS_2_ at a temperature of 700°C, slightly above the flux melting point of 681°C (Vasilyeva and Belaya, 1999). A KBr flux allowed for the crystallization of SmTe_1.80_ single crystals from a mixture of the elements; however, no efforts were made to improve upon the relatively low 5% yield of the phase (Ijjaali and Ibers, 2006). As a final example, it was determined that SnS could be recrystallized as a single phase from a KI flux at 740°C (Timmo et al., 2016).

### Mixed Chalcogenides

The ABI fluxes can offer a convenient reaction media to obtain mixed chalcogenide phases, such as Nd_2_S_2_Te, Dy_4_S_4_Te_3_, CsTb_3_STe_4_, and CsTb_5_S_2_Te_6_. The latter two compounds were crystallized from a CsBr flux at 850°C via the oxidation of Tb metal with S and Te (Lissner et al., 2019). At the same temperature, the direct reaction of Nd, Se, and Te in a stoichiometric ratio resulted in Nd_2_S_2_Te powder, which can be recrystallized in an equimolar amount of CsBr flux (Schleid and Klein, 2001). A ternary mixed chalcogenide phase Dy_4_Se_4_Te_3_ has been obtained starting with Dy_2_O_3_ and B as a reducing agent in a KI flux (Guo and Guo, 2014). A similar composition with very similar unit cell metrics, Dy_4_S_4_Te_2.3_, was obtained by the reaction between Dy_2_S_3_, Dy_2_Te_3_, and Te.

### Ternaries

A number of ternary phases have been obtained from the ABI fluxes. For example, colorless single crystals of Li_3_AsS_3_ were grown from a LiI flux starting with the elements. Although the melting point of LiI is about 469°C, the crystals were successfully grown at 450°C, indicating a possible reaction between the flux and the starting materials led to a melting point lowering of the flux (Huber et al., 2012). A reactive NaBr flux at 950°C was used to produce single crystals of NaIn_3_S_5_ from CaS and In_2_S_3_ (Zeng et al., 2015). In this reaction the flux provides both a reaction medium and functions as the Na^+^ source. Another antimony ternary chalcogenide, Ni_2_SbTe_2_, was obtained by reacting stoichiometric ratios of the elements in a KI flux at 900°C (Reynolds et al., 2004). The same reaction in the absence of the flux resulted in a product with the same composition, however in the form of smaller single crystals. Babo et al. reported on the synthesis of the ternary tellurides RbSc_5_Te_8_, KYTe_2_, and RbYTe_2_ that were prepared using KBr and RbBr as reactive fluxes at 900°C (Babo and Schleid, 2008, 2009). A study on the crystal growth of photoluminescent Pb_2_P_2_S_6_ in KI at 800°C and LiBr/KBr at 500°C fluxes revealed different particle morphologies of the final products as a function of the flux used (Zhang et al., 2014). This serves as a good illustration of how different flux choices (and reaction temperatures) can be used to tune particle size and optical properties of the resulting material.

**Figure 6:**
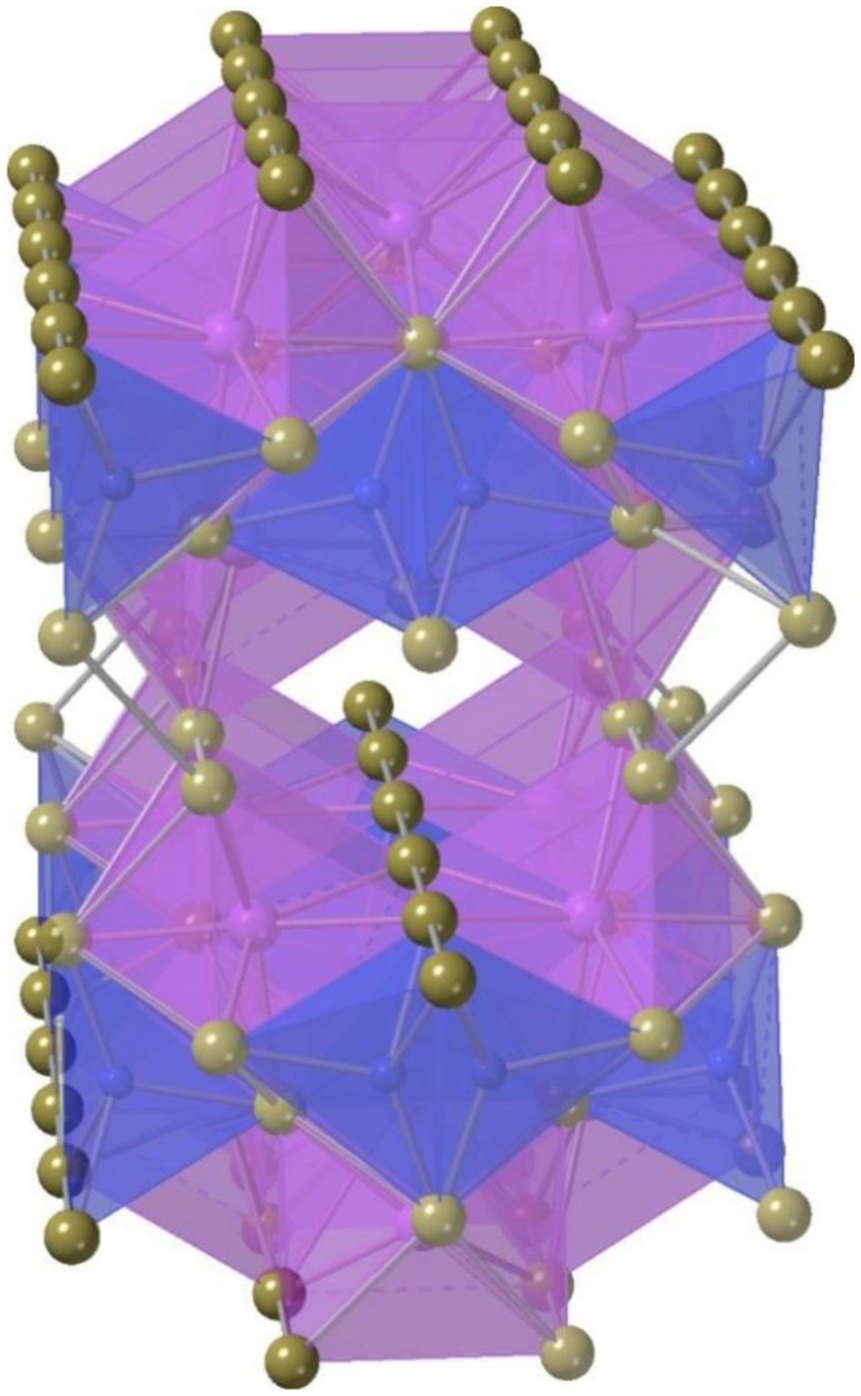
A fragment of Gd_3_Cu_2_Te_7_ structure showing linear Te-Te-Te chains (Huang and Ibers, 2001). Tellurium, gadolinium, and copper are shown in brown, blue, and magenta, respectively.

There are several lanthanide chalcogenide systems that were explored using ABI fluxes (Prakash et al., 2016), and the synthesis of new *f*-element chalcogenides tends to attract a lot of attention because of their interesting structural features and magnetic and optical properties (Bugaris and Ibers, 2010). For example, the structures of Gd_3_Cu_2_Te_7_ and U_2_Cu_0.78_Te_6_, which were both isolated from a KI flux at 850°C, contain linear Te-Te chains (Figure 6), a common structural motif in chalcogenide chemistry (Huang and Ibers, 2001). Numerous compositions can be obtained from ABI fluxes, for example, a large family of lanthanide copper sulfides, *Ln*CuQ_2_ (*Ln* = lanthanides, Q = S, Se), was synthesized using several different flux systems. Successful crystal growth in this system was achieved with CsCl, KBr, and KI fluxes (Ijjaali et al., 2004; Strobel et al., 2005; Strobel and Schleid, 2007), where, interestingly, KBr and KI are interchangeable for the recrystallization of *Ln*CuSe_2_ (La, Ce, Pr, Nd, and Sm) (Ijjaali et al., 2004). An isotypic series of composition RbLnSe_2_ was prepared from a RbI flux using an excess of Rb_2_S_3_, which potentially also functioned as an auxiliary flux. Most *f*-elements are magnetic and due to the localized nature of the 4*f* electrons, RbCeSe_2_, RbTbSe_2_, and RbErSe_2_ exhibit simple paramagnetic behavior with no apparent magnetic interaction between the lanthanide cations. A Curie-Weiss law fit for RbCeSe_2_ shows a large negative Weiss constant, which was attributed to crystal-field splitting of the ^2^F_5/2_ Ce(III) ground state (Deng et al., 2002).

The ABI fluxes allow for the synthesis of compounds containing both 3*d* and 4*f* elements as well as interlanthanide phases, which can result in interesting physical properties due to interactions between the metal cations. For example, a series of compounds Ln_3_TSe_6_ (Ln = Sm, Gd; T = In, Cr) and Tb_3_CrSe_6_ were obtained by reacting stoichiometric quantities of the elements in a KBr flux at 850°C (Tougait and Ibers, 2000a). The products exhibit magnetic properties and magnetic measurements on Gd_3_CrSe_6_ revealed a sharp drop in the magnetic susceptibility below 10(1) K, indicative of an antiferromagnetic transition. No such drop in the susceptibility was observed for the analogous Sm_3_CrSe_6_ and Tb_3_CrSe_6_ compositions, suggesting that the ordering originates from the Gd^3+^ cations. Related mixed 4*f* element chalcogenides, Er_3_Sm*Q*_6_ (*Q* = S, Se) and Er_1.12_Sm_0.88_Se_3_, were grown from a KI flux (Gray et al., 2007). Although the structures of Er_3_Sm*Q*_6_ (*Q* = S, Se) exhibit minimal Er/Sm disorder as they are found in different coordination environments with coordination numbers of 6 and 7 for Er and 8 for Sm, it is difficult to precisely quantify the amount of disorder as their X-ray scattering is very similar.

### Quaternaries

The ABI fluxes often provide alkali cations that incorporate into the final products, thus playing the role of a reactive flux. This incorporation favors more complex compositions that usually result in more complex crystal structures. A number of non-centrosymmetric quaternary chalcogenide phases have been reported and were investigated for their non-linear optical properties. For example, by exploiting the lone pair effect of Sb^3+^ to induce non-centrosymmetry, La_2_CuSbS_5_ was synthesized in a mixed CsBr/BaCl_2_ flux at 900°C (Lin et al., 2019). This compound exhibits a reasonable SHG response (0.5 × AgGaS_2_) with a large LIDT (6.7 × AgGaS_2_). Another couple of NLO active sulfides, Ba_4_CuGa_5_*Q*_12_ (*Q* = S, Se), were grown from a KBr flux at 750°C (Kuo et al., 2013), and their SHG response demonstrated that the sulfide compound is a promising NLO material in infrared region. Further improvements of the optical characteristics were observed in Ba_10_Zn_7_*M*_6_*Q*_26_ (*M* = In, Ga; *Q* = S, Se) that were obtained by a reaction of the elements in a KBr/CsBr flux confined in a graphite crucible at 1,000°C (Li et al., 2018). Similarly, single crystals of Ba_6_Zn_6_ZrS_14_ were grown by the direct combination of the elements in the presence of a KI flux at 900°C (Bezuidenhout et al., 2014). The product mixture contained Ba_2_ZnS_3_ as a side product, and optimization of the solid state reaction condition was able to yield a phase pure sample starting from the elements. The product is stable against water exposure and exhibits good photoluminescent properties that make it a possible candidate for optical applications.

Quaternary lanthanide chalcogenides have been studied extensively for their magnetic properties and several series of compounds have been crystallized out of ABI fluxes. Yao et al. prepared KLn_2_CuS_4_ (Ln = Y, Nd, Sm, Tb, Ho) and K_2_Ln_4_Cu_4_S_9_ (Ln = Dy, Ho) by the reactive flux method using K_2_S and KI to aid crystal growth at 700°C (Yao et al., 2003). All compounds can also be obtained in the absence of KI, however with significantly lower yields. Magnetic measurements performed on K_2_Ho_4_Cu_4_S_9_ showed a paramagnetic behavior with a magnetic moment that agrees well with the theoretical value of 10.60 μ_B_ for Ho^3+^. Two other copper-containing phases, SrCuCeSe_3_ and SrCuPrSe_3_, were obtained from CsI flux at 800°C (Strobel and Schleid, 2004). Despite the very similar sizes of the lanthanide cations in these two compounds, they adopt distinct structure types.

The use of a KBr/BaBr_2_ mixed flux enables Ba incorporation into the final product as can be illustrated by the synthesis of the quaternary chalcogenides Ba*LnMQ*_3_ (*Ln* = lanthanide, *M* = Cu, Ag, Au, *Q* = Se or Te) (Yang and Ibers, 1999). The *Ln* and *M* elements along with Ba*Q* were mixed with a KBr/BaBr_2_ flux and reacted at 850°C. The flux-assisted reactions yielded single crystals in 35–65% yield with minor quantities of *Ln*_2_*Q*_3_ impurities, while only binaries of *Ln*/Te formed in similar reactions without a flux. Inverse magnetic susceptibility data for BaNdAgTe_3_ were fitted to a Curie-Weiss law, and exhibit no magnetic transitions down to 5 K. In a similar way, Ba_4_Nd_2_Cd_3_Se_10_ and Ba_4_*Ln*_2_Cd_3_S_10_ (*Ln* = Sm, Gd, Tb) were formed from a BaBr_2_/KBr flux at 900°C (Yang and Ibers, 2000). The reciprocal magnetic susceptibility of the Tb-containing compound was fit to the Curie-Weiss law and resulted in an effective magnetic moment of 9.94 μ_B_, which is consistent with the theoretical value of 9.72 μ_B_.

The KBr flux offers a convenient way to synthesize pentanary KCaEr_2_CuS_5_, providing both solution for crystal growth and the K^+^ cation that incorporates into the structure (Zeng et al., 2006). Initially, a mixture of Er_2_S_3_, CaS, Cu, and S were employed as starting materials. However, while optimizing the reaction conditions it was determined that an optimal yield is obtained when Cu_2_S is used instead of a mixture of the elements. The compound exhibits paramagnetic behavior down to 5 K and an estimated bandgap of 2.4 eV measured by UV-vis spectroscopy.

### Thiophosphates and Thiosilicates

The ABI fluxes have been used to prepare several families of thiophosphate compounds. Isostructural K_0.5_Ag_0.5_Nb_2_PS_10_ and Rb_0.38_Ag_0.5_Nb_2_PS_10_ were grown from the elements in eutectic AgI/KI and AgI/RbI fluxes, respectively, at 800°C (Dong et al., 2005; Dong Y. et al., 2005). Similarly, KBr and CsBr fluxes at 950°C were used to obtain Rb_3_*Ln*_3_[PS_4_]_4_ (*Ln* = Pr, Er) and Cs_3_Pr_5_[PS_4_]_6_ (Komm and Schleid, 2004, 2005). Crystal growth of rare earth sulfides Na*Ln*P_2_S_6_ (*Ln* = La and Ce) and CsLnP_2_S_7_ (Ln = Pr, Nd, Sm, Gd, Tb, Dy, Ho, Er, Yb, and Y) was facilitated by a CsI/NaI flux at 500°C (Klepov et al., 2019a). The synthesis of the Na*Ln*P_2_S_6_ compounds is preceded by reduction of the P(V) containing P_2_S_5_ starting material to P(IV) in P_2_${\text{S}}_{6}^{4-}$. Possible reducing agents for this reaction are iodide and sulfide anions, although it is unclear which anion contributes more. Magnetic susceptibility measurements revealed paramagnetic behavior of all compounds and the effective moments derived from Curie-Weiss law fits are in a good agreement with the expected theoretical values. In a similar way, uranium thiophosphates were grown from eutectic CsI/NaI and RbI/NaI fluxes from US_2_, Na_2_S, and P_2_S_5_ binaries at temperatures between 500 and 750°C (Klepov and zur Loye, 2018). All compounds exhibit rather complex compositions, Cs_5_Na_6_[U(PS_4_)_4_](PS_4_), Rb_5_Na_3_[U(PS_4_)_4_], CsNa[U(PS_4_)_2_], Cs_1.67_Na_0.52_I_0.19_[U(PS_4_)_2_], Cs_1.033_Na_1.343_I_0.376_[U(PS_4_)_2_], and Rb_1.35_Na_0.93_I_0.28_[U(PS_4_)_2_], and structures. Magnetic susceptibility data for CsNa[U(PS_4_)_2_] indicate the transition from one paramagnetic state to another at 200–225 K, which is not accompanied with a structural change and is likely due to an electronic phenomenon.

There are several thiosilicates that were obtained from ABI fluxes. The serendipitous crystal growth of NaY_3_S_3_[SiS_4_] occurred when Be, Y, and S were reacted in an excess of a NaBr flux inside an evacuated silica tube, which served as a source of Si (Hartenbach and Schleid, 2003). The reaction was then optimized by using SiS_2_ as a starting material in the same NaBr flux at 850°C, which resulted in a water- and air-resistant product in an approximate yield of 80%. Uranium(IV) thiosilicates Cs_2_Na_4_[U_2_(SiS_4_)_2_(Si_2_S_8_)] and Cs_2.12_Na_3.88_[U_2_(SiS_4_)_2_(Si_2_S_7_)] can be obtained from CsI and CsI/NaI eutectic fluxes by reacting US_2_, SiS_2_, and Na_2_S (Klepov et al., 2019b). The reaction yielded phase pure Cs_2_Na_4_[U_2_(SiS_4_)_2_(Si_2_S_8_)], which exhibits paramagnetic properties as revealed by magnetic susceptibility measurements.

## Oxychalcogenides

The ABI fluxes have also been employed to synthesize a variety of oxychalcogenide compounds. These are predominately oxyselenides (Tougait and Ibers, 2000b,c, 2001; Nitsche et al., 2014; Liu et al., 2015; Peschke et al., 2015, 2017a,b; Peschke and Johrendt, 2016, 2017), but also include oxysulfides (Zeng et al., 1999, 2002; Huang et al., 2000; Chi et al., 2016), and oxytellurides (Liu et al., 2007). In many cases, the ABI flux is utilized as a recrystallizing agent. For example, single crystals of Ln_3.67_Ti_2_O_3_Se_6_ (Ln = Ce, Nd, Sm) were grown by mixing a powdered sample of the material, synthesized from stoichiometric quantities of Ln, TiO_2_, Ti, and Se, with KBr in a fused silica tube. The reaction was heated to 950°C for 96 h before being slow cooled to room temperature (Tougait and Ibers, 2000c). In other cases, the materials are synthesized via the traditional flux method, where a combination of metal oxides and metal chalcogenides and/or elemental species, are combined with an ABI flux in an evacuated fused silica tube. La_5_Cu_6_O_4_S_7_ was grown in this way from a mixture of La_2_S_3_, CuO, and KI flux heated to 900°C for 4 d and slow cooled to 300°C at a rate of 3°C/h (Huang et al., 2000). Similarly, La_6_Ti_3_O_5_Se_9_ was grown from a mixture of La, Ti, TiO_2_, Se, and KI flux heated to 950°C for 100 h and slow cooled to 700°C at a rate of 3°C/h (Tougait and Ibers, 2001).

In most cases, the oxychalcogenide material is formed by adding a limited, typically stoichiometric, amount of oxygen, in the form of a metal oxide precursor, into the evacuated fused silica tube. However, in the case of Ln_4_S_3_Si_2_O_7_, the oxygen was obtained from the reaction attacking the fused silica tube (Zeng et al., 1999, 2002). These reactions utilized a KBr flux, as opposed to the alkali iodides that are used for the synthesis of most of the other oxychalcogenide systems, which is the likely cause of the fused silica etching. In a few other cases, an overstoichiometry of oxygen is used in combination with boron metal as an oxygen getter. Fox example, BaGeOSe_2_ was synthesized from a mixture of Ba, GeO_2_, Se, B, and KI flux. In this reaction, the excess oxygen associated with the GeO_2_ is captured by the boron metal to form B_2_O_3_ as a side product (Liu et al., 2015).

The synthesized oxychalcogenides have been explored for two primary purposes. Due to the different size and electronegativity of oxygen vs. chalcogenides, cationic oxychalcogenide polyhedra possess a larger polarity than their pure oxide counterparts. This can lead to increased second-harmonic generation when the oxychalcogenide crystallizes in a non-centrosymmetric space group. This is evident in non-centrosymmetric BaGeOSe_2_, whose GeO_2_Se_2_ and BaOSe_6_ polyhedra have dipole moments with magnitude 11.25 and 20.47, respectively. These are considerably greater than the moments of 4.65 and 4.70 for the respective pure oxide polyhedra and result in BaGeOSe_2_ having a similar strength SHG response as AgGaS_2_, a commercially used NLO crystal (Liu et al., 2015). A second interest in the oxychalcogenides arose from the discovery of high temperature superconductivity in the iron arsenides. As a result, considerable attention has been paid to structurally related rare-earth transition metal oxychalcogenides, for example, RE_2_FeSe_2_O_2_ (RE = La, Ce) (Nitsche et al., 2014).

## Pnictides

### Ternaries

**Figure 7:**
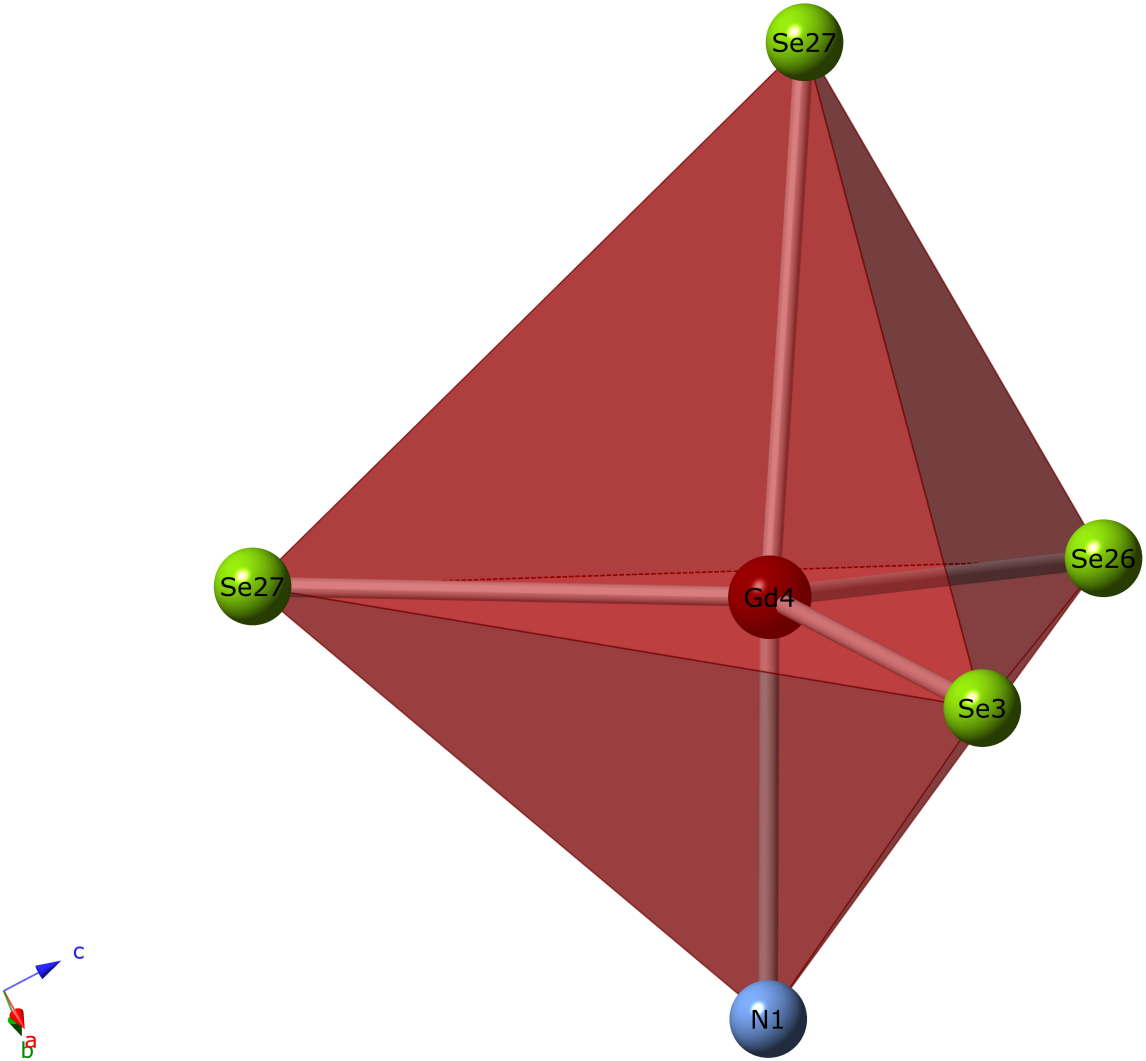
The unusually low 5-fold coordination of Gd(4) in Gd_23_N_5_Se_27_.

The use of ABI fluxes has also resulted in a number of pnictide phases exhibiting unique structural features, further demonstrating the versatility of the alkali bromide and iodide flux systems. As an example, Lissner and Schleid employed a CsBr flux in reactions of lanthanide metal powders, sulfur, cesium azide, and lanthanide tribromides at 900°C to obtain single crystals of Ln_4_N_2_S_3_ (Ln = La-Nd) (Lissner and Schleid, 2006, 2017). It was noted that the four structures, which are isotypic to one another, exhibit an elongated Ln-S bond and an increase in the Ln(2) site coordination number from 6 to 6+1 along the series from La to Ce, an observation which diverges from expectations based on the lanthanide contraction. The authors later used similar methods to obtain the Ln_3_NS_3_ (Ln = La-Nd, Sm, Gd-Dy) series from a NaBr flux (Lissner et al., 2006). Schurz et al. obtained the first gadolinium nitride selenides, Gd_3_NSe_3_ and Gd_23_N_5_Se_27_, by replacing sulfur with selenium and using gadolinium triiodide with a CsI flux (Schurz et al., 2013). A distinctive feature in the structure of Gd_23_N_5_Se_27_ is the presence of gadolinium cations with a coordination number of 5, significantly lower in coordination than what is typically observed in lanthanide solid-state structures (Figure 7), which was attributed to the short coordinating anion bond distances of 2.20 Å for Gd(4)-N(1) and 2.77 Å for Gd(4)-Se(27).

In an effort to prepare new low-dimensional materials for semiconductor applications, Na_2_BP_2_, a novel boron phosphide containing one-dimensional B-P chains was prepared (Woo et al., 2019). To avoid formation of inert and thermodynamically-favored phases, Woo et al. utilized mild reaction temperatures and oxidative elimination to prepare single crystals of the metastable Na_2_BP_2_ phase from a mixture of Na_3_BP_2_ and CuI in a CsI-NaI eutectic that was heated to 500°C. The authors suggested that this method may also offer a route to multidimensional materials by increasing the degree of oxidation. Using similar methods, Aydemir et al. serendipitously obtained Eu_3_[B_3_N_6_], the first entirely europium(III) nitridoborate (Aydemir et al., 2016). This phase was initially prepared from a NaBr flux and formed as a minor phase due to an EuBr_3_ impurity in the starting materials; it was surmised that the decomposition of EuBr_3_ into EuBr_2_ and Br_2_ resulted in the oxidation step which yielded the product. This hypothesis was confirmed by heating a mixture of a divalent europium precursor Eu_3_[BN_2_]_2_ and Br_2_ to 800°C, yielding a mixture of Eu_3_[B_3_N_6_] and EuBr_2_ (Aydemir et al., 2016).

### Quaternaries

As opposed to the ternary pnictides, there have been only a few reports on the use of ABI fluxes to prepare quaternary pnictide phases. While combinations of lanthanide metal powder, sulfur or selenium, cesium azide, and lanthanum tribromides resulted in several ternary phases [Ln_4_N_2_S_3_ (Ln = La-Nd), Gd_3_NSe_3_, and Gd_23_N_5_Se_27_], replacement of cesium azide with sodium azide resulted in the quaternary phase Nd_3_N_2_SeBr (Lissner and Schleid, 2007), an outcome that emphasizes the importance in the choice of starting materials.

For photoelectrochemical water splitting applications, particles of SrNbO_2_N were pre-calcined and heated in a variety of alkali halide fluxes; it was determined that SrNbO_2_N particles flux-treated in NaI yielded the highest photocurrent density of 1.5 mA cm^−2^ at 1.23 V_RHE_ under AM 1.5 G irradiation (Kodera et al., 2016). In the search for potential superconductors, LnFeAsO (Ln = La-Nd, Sm, Gd-Tb) has been studied, and was prepared by Nitsche et al. from a mixture of FeO, As, and lanthanide metal in a NaI-KI flux at 1,050°C (Nitsche et al., 2010). Similar methods were later used to produce large single crystals of LaFeAsO from a KI flux, which were isolated as plates having an edge length of up to 1 mm (Jesche et al., 2012).

## Salt-Inclusion Materials

Salt-inclusion materials (SIMs) represent a unique class of hierarchical materials that typically exhibit porous and covalent frameworks with interpenetrating ionic salt lattices. As functional materials, SIMs have wide-ranging applications as waste storage and ion exchange materials to optical materials. The zur Loye group has focused on synthesizing novel uranium-containing SIMs via alkali halide fluxes and while the majority of these have been prepared from ACl-AF eutectics, some success was achieved with the use of ABI fluxes. A novel intergrowth uranyl silicate, K_8_(K_5_F)U_6_Si_8_O_40_, was grown from a KF-KBr flux (Morrison et al., 2016b), and unlike many other uranyl compounds, K_8_(K_5_F)U_6_Si_8_O_40_ was found to exhibit intense luminescence behavior possibly attributable to the salt-inclusion. Similarly, a KF-KBr flux was used to prepare [KK_6_Br_0.6_F_0.4_][(UO_2_)_3_(Ge_2_O_7_)_2_] (Juillerat et al., 2018) in a study to explore the adaptability of the uranyl germanate SIM framework. This [(UO_2_)_3_(Ge_2_O_7_)_2_] framework was found to successfully accommodate twelve different salt-inclusions that were incorporated based on the selected flux used in the synthesis.

**Figure 8:**
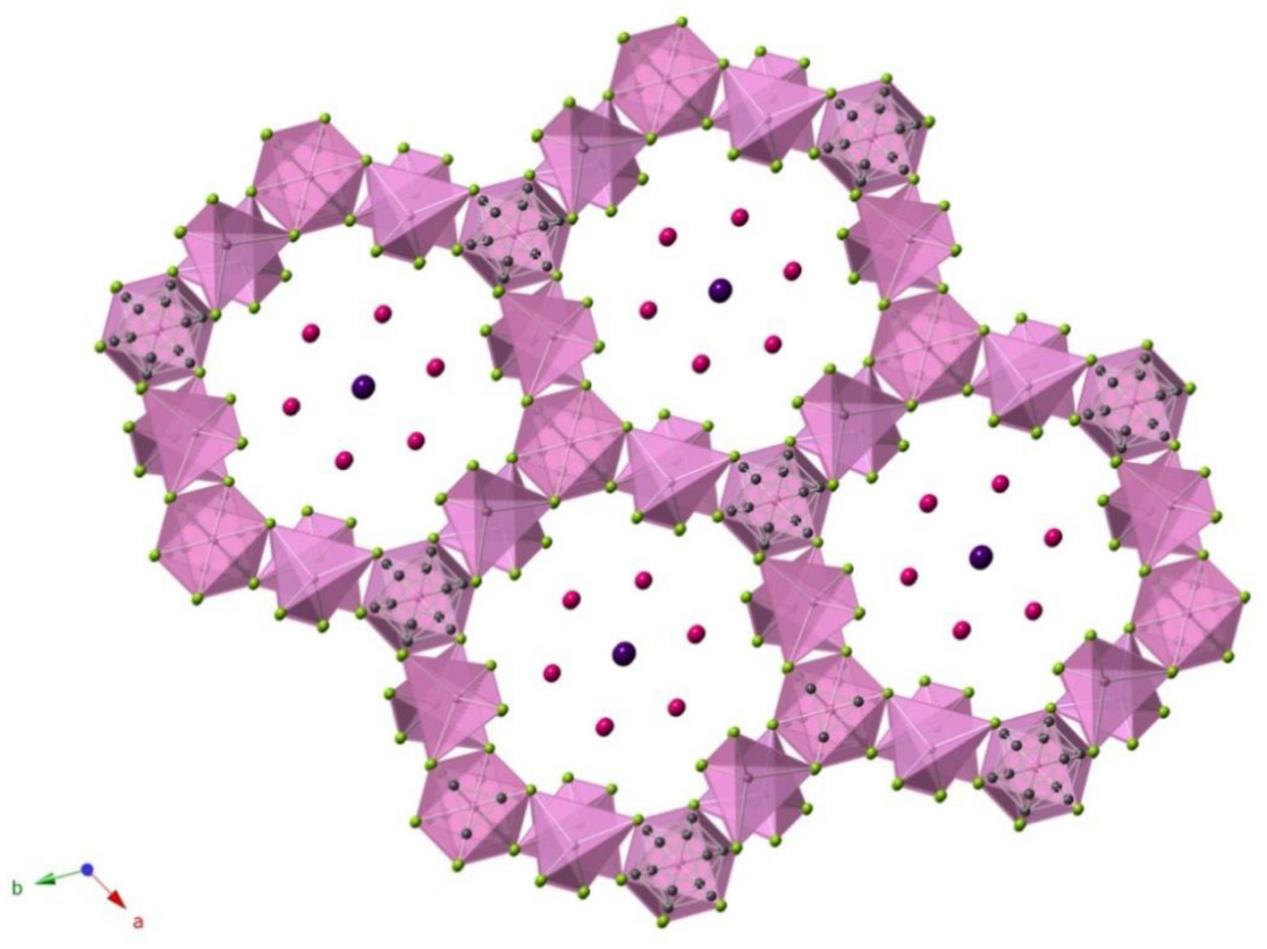
A view on the structure of (K_3_I)[InB_12_(InSe_4_)_3_] salt-inclusion compound, which was obtained using KI flux (Guo et al., 2016).

In addition to uranium-containing SIMs, ABI fluxes have demonstrated moderate success in stabilizing non-uranium SIMs as well. For example, the pentanary selenide (K_3_I)[InB_12_(InSe_4_)_3_] was synthesized from a KI flux (Guo et al., 2016). The crystal structure of (K_3_I)[InB_12_(InSe_4_)_3_] is non-centrosymmetric and was found to be SHG active (Figure 8), exhibiting a higher SHG intensity than the isostructural (K_3_I)[SmB_12_(GaS_4_)_3_], which the authors suggested was on account of the larger dipole moment of the InSe_4_ unit relative to GaS_4_.

## Nanomaterials

While flux crystal growth is typically used for the growth of single-crystal X-ray diffraction or larger sized single crystals (i.e., >50 μm), ABI fluxes have also been used successfully in the preparation of nanostructure materials. For example, upon co-precipitating precursor FeCo particles, Kishimoto et al. facilitated particle growth by heating at 850°C in a KBr flux (Kishimoto et al., 2017). For applications in photocatalysis, silver-loaded sodium titanates were prepared by traditional solid-state and flux methods, and while several ABI fluxes were evaluated including NaBr and NaI, it was determined that the Ag/Na_2_Ti_6_O_13_ prepared from a NaCl flux resulted in the highest CO formation rate and the highest selectivity to CO (Zhu et al., 2019). Kong et al. determined that Fe_7_S_8_ nanorods and nanosheets could be prepared from a KI flux and that the morphology of the nanostructures was dependent upon the reaction temperature, where nanorod formation was favored at 750°C and nanosheets at 850°C (Kong et al., 2005).

**Figure 9:**
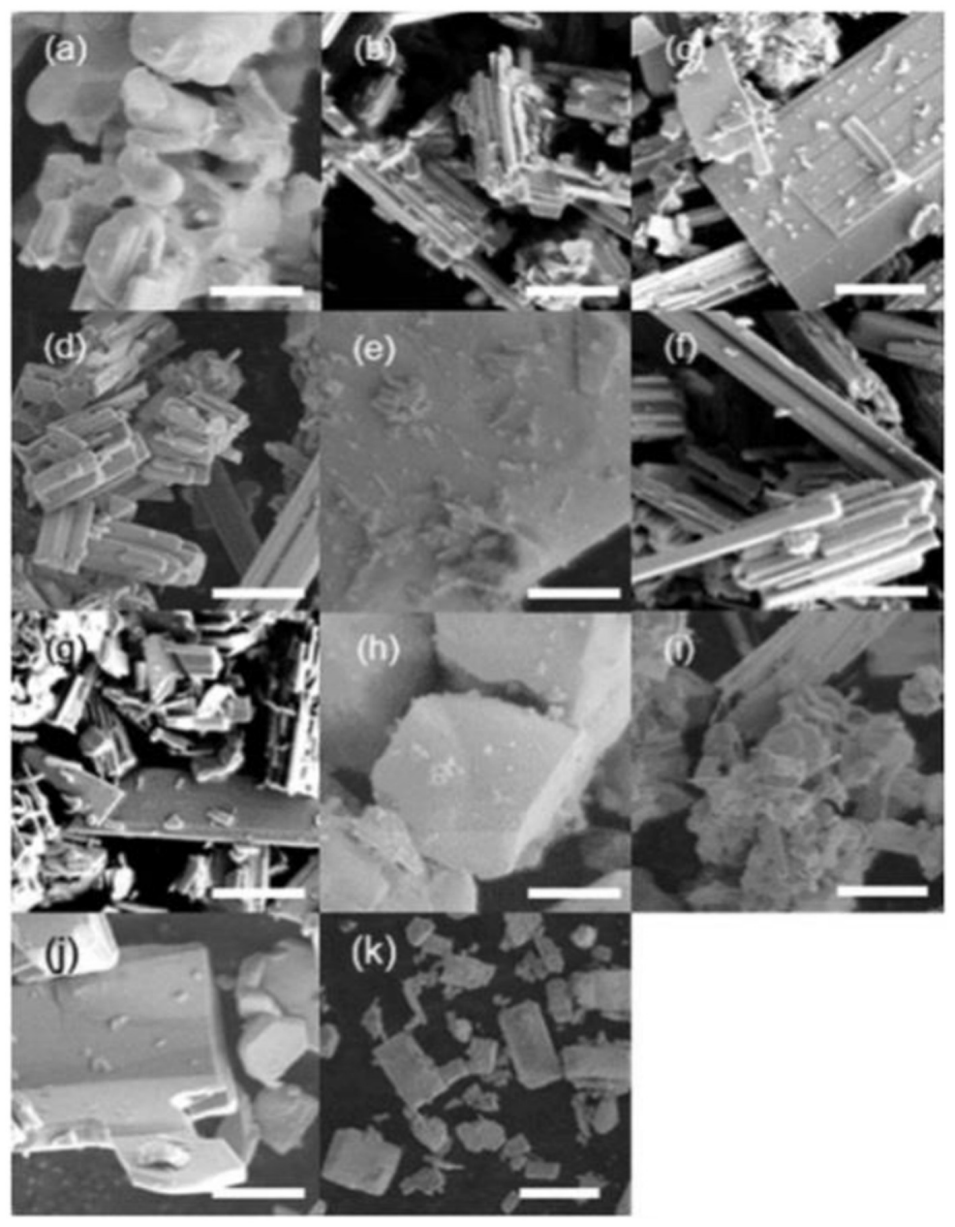
SEM images of oxide precursors produced **(a)** without flux, and with **(b)** NaCl, **(c)** KCl, **(d)** RbCl, **(e)** SrCl_2_, **(f)** NaBr, **(g)** KBr, **(h)** NaI, **(i)** KI, **(j)** CsI, and **(k)** SrCl_2_ fluxes. The scale bars in **(a–j)** are 2 μm, and that in **(k)** is 20 μm. Reproduced from Kodera et al. (2016) under the Creative Commons CCBY-NC license—published by the Royal Society of Chemistry.

For applications in photoelectrochemical water splitting, Kodera et al. also found that the choice of flux and reaction conditions strongly affected the morphology of SrNbO_2_N particles (Kodera et al., 2016): for example, the use of a NaBr flux resulted in column-like morphologies, while plate morphologies resulted from KBr flux, and large block-like morphologies were resultant from NaI flux (Figure 9). The best photocurrent density of 1.5 mA cm^−2^ at 1.23 V_RHE_ under AM 1.5 G irradiation was achieved using a NaI flux with pre-calcination. Similarly, in their search for efficient light harvesting and charge transport materials Shi et al. determined that a KI flux could be used to prepare Ta_3_N_5_ nanorod arrays that were highly oriented along the [100] direction, where the size of the nanorods was controlled by the amount of flux present in the reaction (Shi et al., 2018).

## Conclusion

The ABI fluxes offer a convenient tool that provides a reaction and crystallization medium for different classes of inorganic compounds, mostly chalcogenides. They can be used in a very broad temperature range, from ~207 to ~1,300°C, by selecting pure salts or their eutectics using phase diagram databases, such as FactSage (Bale et al., 2002, 2009, 2016). Both the selection of flux and the temperature profile can significantly affect the size and the shape of the resulting crystals, and often the reaction conditions have to be thoroughly optimized to achieve crystals of XRD quality. The ABI fluxes are not very reactive toward common reaction vessel materials, such as silica and alumina, which enables their broad use with different types of crucibles. ABI fluxes can be easily separated from the reaction products using water, methanol, DMF, and other polar solvents. Most commonly, the elements and binaries are used as starting materials for ABI flux crystal growth; however, several new approaches coupled with the use of ABI fluxes have been developed recently. One of them, a reaction of an oxide with elemental boron and sulfur, shows a good promise for the synthesis of new chalcogenides as it allows using available oxide precursors and binds oxygen in the system by the excess of boron, which can result in purer products, especially for the elements that are known for binding oxygen, such as uranium and lanthanides.

As ABI fluxes provide a versatile platform for the synthesis of new compounds and different morphologies (crystals or nanoparticles), they will continue to be used for the design of new materials to meet the demands of modern technology. Alkali halide fluxes have been proven useful for the crystal growth of quantum spin liquid candidates (Ferreira et al., 2020), and ABI fluxes can serve as a facile route toward new magnetic and optical materials containing soft anions, such as chalcogenides. Further control over the properties of the new materials can be achieved by using ABI fluxes for design of particle size and shape, to create novel classes of nanomaterials. Therefore, the ABI flux has been established as a method of synthesis, and the future research will focus on using ABI fluxes for the synthesis of new classes of materials.

## Author Contributions

HzL proposed the idea of the manuscript and participated in writing the manuscript. VK, CJ, KP, and GM contributed to the literature search and writing the manuscript. All authors contributed to the article and approved the submitted version.

## Conflict of Interest

The authors declare that the research was conducted in the absence of any commercial or financial relationships that could be construed as a potential conflict of interest.
